# Retention in care among HIV-infected pregnant and breastfeeding women on lifelong antiretroviral therapy in Uganda: A retrospective cohort study

**DOI:** 10.1371/journal.pone.0187605

**Published:** 2017-12-22

**Authors:** Simon Muhumuza, Evelyn Akello, Charity Kyomugisha-Nuwagaba, Rose Baryamutuma, Isaac Sebuliba, Ibrahim M. Lutalo, Edgar Kansiime, Linda N. Kisaakye, Agnes N. Kiragga, Rachel King, William Bazeyo, Christina Lindan

**Affiliations:** 1 School of Public Health, Makerere University, Kampala, Uganda; 2 AIDS Control Program, Ministry of Health, Kampala, Uganda; 3 Infectious Disease Institute, School of Medicine, Makerere University, Kampala, Uganda; 4 Global Health Sciences, University of California San Francisco, San Francisco, California, United States of America; Azienda Ospedaliera Universitaria di Perugia, ITALY

## Abstract

**Background:**

In 2013, Uganda updated its prevention of maternal-to-child transmission of HIV program to Option B+, which requires that all HIV-infected pregnant and breastfeeding women be started on lifelong antiretroviral therapy (ART) regardless of CD4 count. We describe retention in care and factors associated with loss to follow-up (LTFU) among women initiated on Option B+ as part of an evaluation of the effectiveness of the national program.

**Methods:**

We conducted a retrospective cohort analysis of data abstracted from records of 2,169 women enrolled on Option B+ between January and March 2013 from a representative sample of 145 health facilities in all 24 districts of the Central region of Uganda. We defined retention as “being alive and receiving ART at the last clinic visit”. We used Kaplan-Meier analysis to estimate retention in care and compared differences between women retained in care and those LTFU using the chi-squared test for dichotomized or categorical variables.

**Results:**

The median follow-up time was 20.2 months (IQR 4.2–22.5). The proportion of women retained in HIV care at 6, 12 and 18 months post-ART initiation was 74.2%, 66.7% and 62.0%, respectively. Retention at 18 months varied significantly by level of health facility and ranged from 70.0% among those seen at hospitals to 56.6% among those seen at lower level health facilities. LTFU was higher among women aged less than 25 years, 59.3% compared to those aged 25 years and above, 40.7% (*p* = 0.02); among those attending care at lower level facilities, 44.0% compared to those attending care at hospitals, 34.1% (*p* = 0.01), and among those who were not tested for CD4 cell count at ART initiation, 69.4% compared to those who were tested, 30.9% (*p* = 0.002).

**Conclusion:**

Retention of women who were initiated on Option B+ during the early phases of roll-out was only moderate, and could undermine the effectiveness of the program. Identifying reasons why women drop out and designing targeted interventions for improved retention should be a priority.

## Introduction

Globally, an estimated 2.6 million children less than five years of age were living with HIV in 2014 [1], 90% of whom were in sub-Saharan Africa [1–3]. Most of these children have been infected through maternal-to-child transmission (MTCT) [4], the risk of which can be as high as 10–30% during pregnancy and breastfeeding if no preventive measures are taken [5, 6]. However, if HIV-infected women begin antiretroviral treatment (ART) during pregnancy and continue throughout breastfeeding, MTCT of HIV can be reduced to less than 5% [4, 6]. In 2013, the World Health Organization (WHO) recommended that countries implement the Option B+ strategy, which includes early HIV testing and provision of lifelong ART to HIV-infected pregnant and breastfeeding women regardless of CD4 count or WHO clinical stage [7]. The goal is to reduce MTCT of HIV to less than 5% at the population level [8]. These recommendations were first adopted by three countries in sub-Saharan Africa: Malawi, Zambia and Uganda.

For Option B+ to be successful, women should be started on treatment immediately after HIV diagnosis [9]. In addition, women must be retained in care and on ART to prevent HIV transmission to the infant during breastfeeding, to ensure receipt of drugs, to evaluate for medication toxicities and to identify treatment failure early [10]. Women who continue in care often receive social support and secondary prevention messages that help them cope with the need to be on ART for life [11]. For these reasons, UNAIDS urged countries to ensure that 90% of those on treatment be retained on ART [12]. Unfortunately, however, poor retention in HIV care has been reported in many countries in Africa [13–15], including Uganda [16–18]. Various reasons for loss to follow-up (LTFU) have been identified, including long distances to health facilities, financial constraints, food shortage and insecurity, drug side effects, stigma, fear of disclosure and inadequate social support [18–20].

Uganda began rolling out Option B+ nationally in 2013. The national guidelines require that all antenatal care clinics provide HIV testing for pregnant women at their first clinic visit, and that women identified as sero-positive be started on ART immediately without waiting for the results of CD4 testing [6]. HIV-infected pregnant women must return regularly for refills of drugs and assessment of adherence.

The Option B+ program was implemented in a phased approach, beginning with health facilities in the central region of the country, because the HIV prevalence among women (11.1%) in that region was higher than the national average of 7.3% [21, 22]. By the end of 2013, Option B+ was being provided by 95% of health facilities nationwide. Here, we describe retention among women initiated on Option B+ during the early phases of program implementation.

## Materials and methods

### Study setting

The study was carried out in all 24 districts of the Central region of Uganda because this made it possible to obtain information on retention among HIV-infected women up to 18 months after they started ART. According to the 2014 national census, the Central region had a total population of 9,579,119 or 27.5% of the entire population of the country [23]. The health care system in Uganda is organized under a hierarchy of health centres (HCs) from HC I-IV, with district and regional referral hospitals. Hospitals, HC IVs and IIIs are accredited to provide ART including Option B+ [24]. At the time of the study, there were 332 health facilities in the central region that provided Option B+ services, including 37 hospitals, 45 HC IVs and 250 HC IIIs (hmis2.health.go.ug).

### Study design and sampling

We performed a retrospective cohort analysis of data abstracted from Ministry of Health (MoH) standardized clinic and facility-based records of HIV-infected pregnant or breastfeeding women enrolled on Option B+ between January and March 2013. There were no exclusion criteria by age or other characteristics. We used a multi-level stratified sampling design to select health facilities. All health facilities in each of the 24 districts in the Central region which provided Option B+ services were enumerated and stratified according to their level of care (HC III, IV and hospital). Based on sample size calculations and knowledge of the size of the clinic population, we systematically sampled about 1/3 of all facilities within each health facility strata in each district. The sampling interval was obtained by dividing the total number of facilities with the number of facilities to be studied in each strata. After obtaining a random start from a table of random numbers, the interval was followed until the required number of facilities within each group of health facilities was identified. All available records of eligible women from each of the selected facilities were reviewed in February 2015.

### Data management and analysis

Trained research assistants abstracted data onto structured paper-based forms; completeness was checked by supervisors on a daily basis. Information included age, place of delivery (health facility vs home), level of health facility, CD4 cell count within one month of ART initiation and monthly visits to the clinic up until 18 months post-ART initiation. Data from the paper abstraction forms were electronically captured in Epi Data software (version 3.1) and exported to STATA 12.0 (Stata Corp., Texas, USA) for analysis. We used descriptive statistics including means (and standard deviations), medians (and interquartile ranges [IQRs]) to describe continuous data, and proportions for categorical data. The main outcome variable was retention of women in care, defined as being alive and receiving ART at the last clinic visit.

Kaplan-Meier survival analysis was used to estimate the proportion of women retained in care at 6, 12 and 18 months after ART initiation. This method is used to estimate survival or follow-up when dealing with differing survival or follow-up times [25]. The survival time, in our case, the duration from ART initiation, was terminated by the event of interest, which was dropping out of care. Women were considered lost to follow-up if they failed to return for the last three clinic visits, equivalent to 90 days, and were not identified as having died or transferred out. This 90-day window is similar to what has been used in other studies from the region [10, 26]. Women for whom data indicated that they had died were censored at their approximate date of death. Differences in the descriptive variables between women retained in care at 18 months and those LTFU were compared using chi-squared test for dichotomized or categorical variables. To adjust for clustering, the chi-squared test statistic was divided by the design effect [27]. We tested for any associations between age, facility level and place of delivery and whether a CD4 count had been performed at the time of ART initiation and found no significant relationship.

### Ethical considerations

The Makerere University School of Public Health Ethics Committee, the Uganda National Council for Science and Technology, and the office of the Associate Director for Science, Division of Global HIV/AIDS, Centers for Disease Control (CDC) and Prevention, Atlanta, all reviewed and approved the protocol. The District Health Offices and the health facilities provided permission to conduct record reviews. The Ethics Committee waived the need for consent since no specific identifying individual information was collected, entered into a data base, or reported upon.

## Results

We abstracted records of 2,169 HIV-positive pregnant and breastfeeding women initiated on ART between January and March 2013. The median follow-up time was 20.2 months (IQR 4.2–22.5). The highest proportion of records (39.3%) were abstracted from HC IIIs. Nearly all mothers (96.5%) were initiated on a tenofovir/lamivudine/efavirenz regimen. The mothers’ age at ART initiation ranged from 15–48 years with a median (IQR) of 25 (22–29) years. Data on whether a CD4 cell count had been performed was missing for 900 (41.5%) women. The proportion of women who had a CD4 cell count test result at ART initiation varied considerably by health facility level; 44.0% at HC III, 53.9% at HC IV and 84.2% at hospital level. The median CD4 cell count at ART initiation, among those who were tested, was 524 cells/μl (IQR 346–736). The intracluster correlation coefficient was 0.12 and the design effect was 1.82.

Overall, 1,219 (56.2%) were alive and retained on ART during the follow-up period; 13 (0.6%) were dead and 866 (39.9%) were lost to follow-up. LTFU was higher among women aged less than 25 years, 59.3% compared to those aged 25 years and above, 40.7% (*p* = 0.02); among those attending care at lower-level facilities 44.0% compared to those attending care at hospitals 34.1% (*p* = 0.01) and among women who were not tested for CD4 cell count at ART initiation, 69.4% compared to those who were tested 30.9% (*p* = 0.002) (Table 1).

**Table 1:** Baseline characteristics of women enrolled on Option B+ between January and March 2013.

Characteristic	Women retained at 18 months (N = 1,291) (%)	Women not retained at 18 months (N = 866) (%)	χ^2^ *p*-value	Log rank *p*-value
**Age at ART initiation**				
≤25 years	624 (46.9)	474 (59.3)	0.02	<0.001
>25 years	707 (53.1)	326 (40.7)		
**Health facility level**				
HC III	487 (36.4)	360 (44.0)	0.01	<0.001
HC IV	290 (21.7)	179 (21.9)		
Hospital	562 (42.0)	279 (34.1)		
**CD4 cell count done at ART initiation**				
Yes	955 (71.3)	253 (30.9)	0.002	0.001
No	384 (28.7)	565 (69.4)		
**CD4 cell count at ART initiation (cells/μl)**^**a1**^				
<500	433 (45.1)	156 (51.3)	0.15	0.07
≥500	527 (54.9)	148 (48.7)		

^**a1**^ CD4 cell count among women who had a CD4 cell count done at ART initiation (n = 1,264).

**Fig 1:**
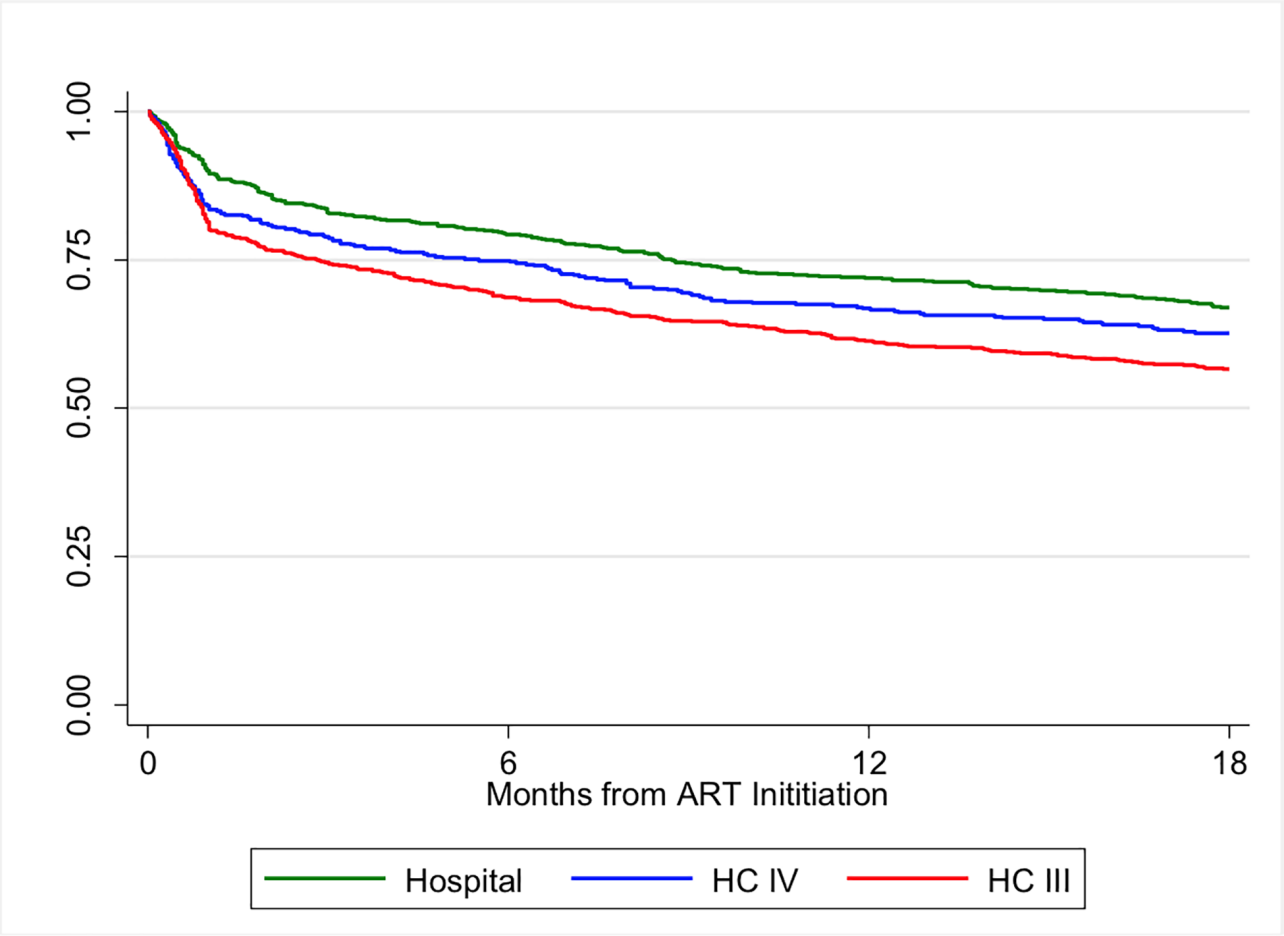
Retention in care over time among women initiated on Option B+ between January and March 2013 in Uganda, stratified by level of health facility.

Based on Kaplan-Meier analysis, the cumulative proportion of women retained in care at 6, 12 and 18 months was 74.2%, 66.7% and 62.0%, respectively (Fig 1).

**Fig 2:**
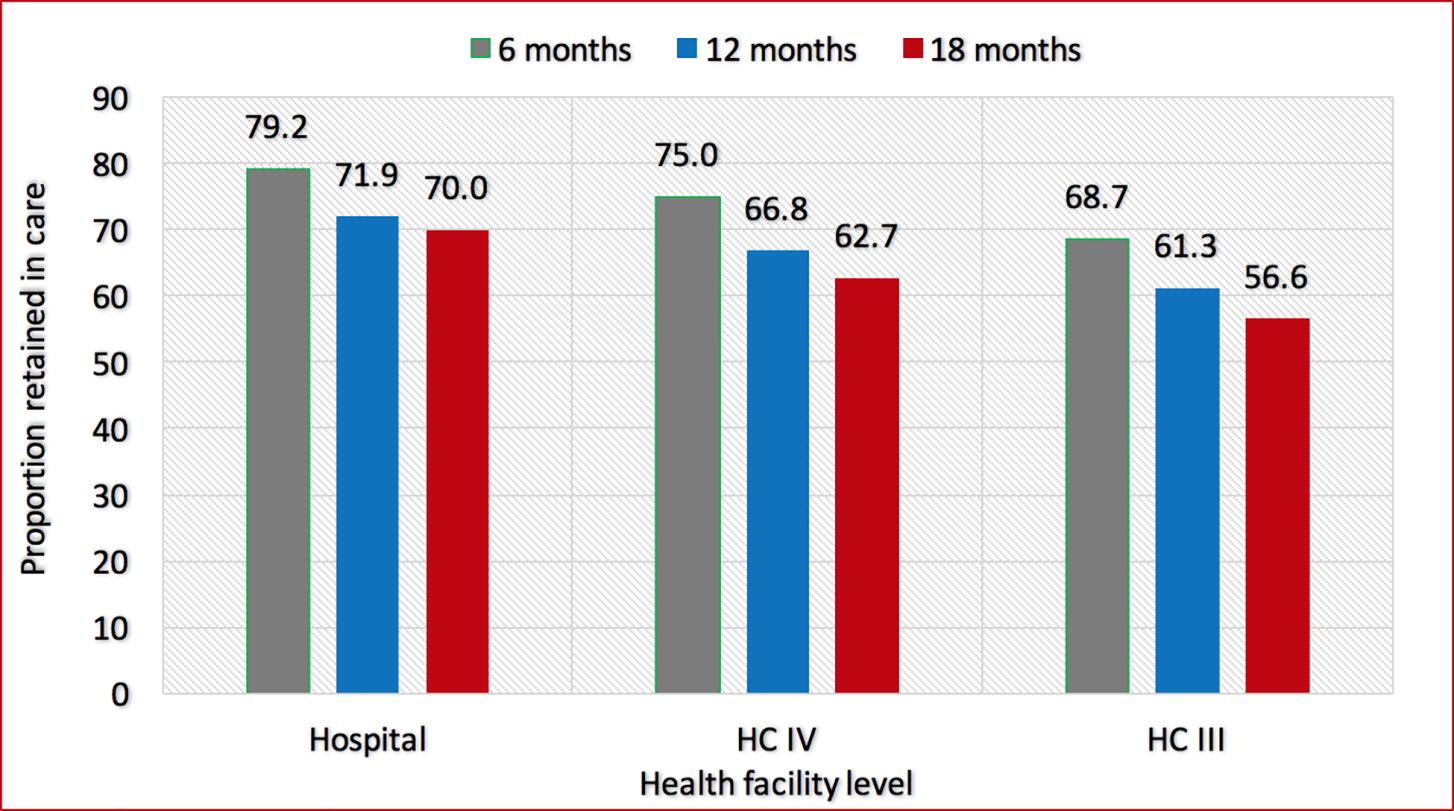
Proportion of women on Option B+ retained in care at 6, 12 and 18 months, over time, by level of health facility.

The greatest rate of drop-out, regardless of type of health facility, occurred during the first three months. Retention at each time point varied significantly by level of health facility and was highest among women seen at hospitals and lowest among those seen at HC IIIs (p<0.001). Among women seen at hospitals, the proportion retained at 6, 12 and 18 months was 79.2%, 71.9% and 70.0%, respectively. For those seen at HC IV, the proportion retained at 6, 12 and 18 months was 75.0%, 66.8% and 62.7%, respectively; and among women seen at HC IIIs, the proportion retained at 6, 12 and 18 months was 68.7%, 61.3% and 56.6%, respectively (Fig 2).

## Discussion

We found that less than two-thirds of women remained in care 18 months following ART initiation, which is below the UNAIDS target of 90% [12]. This level of retention is lower than among clients attending ART clinics in Uganda, as well as in other east African countries where more than 80% of adults remained in care up to 2.5 years following treatment initiation [28, 29]. It is also lower than that reported by national prevention of mother to child transmission (PMTCT) of HIV programs in Malawi (80%) [15, 30] and Ethiopia (83%) [26], although the definitions used to describe LTFU vary across programs [11].

Shortly after the time period during which mothers were evaluated in this study, the MoH instituted several measures to improve follow-up, including scheduling clinic visits for both mothers and HIV-exposed infants on the same days. In addition, mothers who missed scheduled appointments were sent text messages, or in some cases, were also visited at home by health care workers or community volunteers to remind them to return to the clinic. Therefore, it is possible that retention of mothers initiated on treatment is currently higher than what we have reported here. Unfortunately, there has been no systematic evaluation of retention since these measures were implemented to determine whether more women remain in care.

Low levels of retention can undermine the desired effectiveness of the Uganda PMTCT program in a number of ways. Failure to become virally suppressed increases the possibility of transmitting HIV to an infant through breastfeeding, and places children at risk of perinatal infection [31]. Women who do not remain on ART may also develop symptomatic HIV disease, transmit HIV to uninfected sexual partners, and develop drug resistance, particularly if they are taking ART intermittently [9, 32]. Thus, it is critical that reasons for poor retention be explored and addressed.

In our evaluation, women receiving care at lower-level health facilities, which are less resourced, were more likely to be LTFU. This finding is in contrast to reports from other resource-limited countries [15, 26, 30] in which women who attended hospitals compared to those at lower-level health facilities were more likely to be lost. In larger hospital-based sites, factors such as patient burden per staff and longer waiting times have been reported to contribute to LTFU [33]. In Uganda, women attending HC IIIs tend to live in rural areas and have fewer economic resources, including money to pay for transportation, which may have contributed to poorer retention among these women in our study. Numerous studies from Uganda have identified barriers to transportation as a reason for LTFU [10, 15, 34], particularly as 20% of the population lives below the poverty line [35]. Among poor families, work and child care responsibilities compete with the cost of and time required to travel to clinics for appointments [36]. In addition, lack of food with which to take the drugs can also be a barrier to retention [37]. When taken on an empty stomach, the side effects of ART are exacerbated [38]. Because some ARV drugs increase one’s appetite [39], women may drop out of the program if they do not have adequate food. In addition, during seasons when food scarcity is greatest, women spend long work days searching for food and may forget to take their medication.

We also found that younger women were less likely to be retained, which is similar to reports of studies conducted in Ethiopia [26], Malawi [15], and Zimbabwe [40]. Younger women may not understand the benefits of taking ART, or have not experienced those benefits, particularly if they are first-time mothers [41]. They may be more susceptible to the consequences of stigma [10] and may not have as much social support as older women [38, 42]. More mature women may be better able to manage taking ART consistently, to disclose their status to spouses and to work through the consequences of doing so [15, 26].

In our analysis, women who had a CD4 cell count test done at ART initiation were more likely to remain in care compared to those who did not. Less than 60% of the women evaluated in this study, particularly those seen at lower level health facilities, had undergone CD4 cell count testing. Although the Ugandan national guidelines for HIV treatment at the time of our study recommended bi-annual immunological monitoring [6], CD4 cell count testing was not a requirement for initiating women on Option B+. Nevertheless, it is possible that receiving the results of CD4 cell count testing may increase one’s awareness of HIV infection, including the benefits of engaging in care [26]. It has been previously reported that a patient’s knowledge of his or her CD4 count can influence retention [43]. Since this study was implemented, viral load (VL) testing has been rolled out and is now the norm for monitoring response to treatment in Uganda, supplanting the need for regular CD4 cell count tests [44]. As such, these findings may no longer apply to care as it is currently provided.

This study has several limitations. First, retention in care was based on the availability of records from the facility at which a woman initially enrolled. It is possible that women lost-to-follow-up may have died or transferred to other facilities without documentation and thus have been misclassified. Unaccounted transfers are common in Uganda [10]. Secondly, this study relied on a retrospective review of records. The information obtained was limited by routinely recorded data. In particular, we were unable to obtain information on basic socio-demographic and clinical characteristics such as marital and educational status, income level, parity and the timing of HIV diagnosis which may have been associated with retention. Finally, routine health facility information does not include actual reasons for LTFU.

## Conclusions

This study demonstrated that during the early phase of Option B+ roll-out in Uganda, retention in care of HIV-infected pregnant women starting treatment was only moderate. Interventions for young HIV-infected pregnant women may need to be different from those that target retention among the general HIV-infected population. These might include providing outreach services, using motivating text messages more widely, systematically tracing those who drop out or transfer to other clinics and working to mitigate stigma and discrimination.

## Supporting information

S1 Appendix Data required to replicate this study can be recovered from S1 Appendix. (XLSX)
